# An Account of the Varioloid Epidemic, Which Has Lately Prevailed in Edinburgh and Other Parts of Scotland: With Observations on the Identity of Chicken-Pox with Modified Small-Pox. In a Letter to Sir James M'Grigor, Director-General of the Army Medical Department

**Published:** 1820-12

**Authors:** 


### CRITICAL ANALYSIS

OF

### RECENT PUBLICATIONS, IN THE DIFFERENT BRANCHES OF MEDICINE AND SURGERY.

---

"I would have men know, that, though I reprehend the easie passing over of the causes of things "by ascribing them to secret and hidden vertues and properties; (for this hath arrested and laid "asleepe all true enquiry and indications;) yet I doe not understand but that, in the practical "part of knowledge, much will be left to experience and probation, whereunto indication cannot "so fully reach: and this not only in *specie*, but in *individuo*. Yet it was well said, *Vere scire* "*esse per causas scire*."—BACON.

---

*An Account of the Varioloid Epidemic, which has lately prevailed in Edinburgh and other Parts of Scotland: with Observations on the Identity of Chicken-Pox with Modified Small-Pox. In a Letter to Sir James M'Grigor, Director-General of the Army Medical Department.* By JOHN THOMSON, M.D. F.R.S.E. Surgeon to the Forces, Honorary Member of the Royal Medical Society of Edinburgh, Professor of Surgery to the Royal College of Surgeons, Regius Professor of Military Surgery in the University, and Consulting Physician to the Edinburgh New-Town Dispensary. 8vo. pp. 322; Appendix, 78. London: Longman and Co. 1820.

*A History of the Variolous Epidemic which occurred in Norwich in the Year* 1819, *and destroyed* 530 *Individuals, with an Estimate of the Protection afforded by Vaccination, and a Review of past and present Opinions upon Chicken-Pox and Modified Small-Pox.* By JOHN CROSS, Member of the Royal College of Surgeons in London, Corresponding Member of the Société Médicale d'Emulation of Paris, late Demonstrator of Anatomy in the University of Dublin, &c. 8vo. pp. 296. London: Burgess and Hill, 1820.

WE had hitherto deferred taking these works into consideration, because we had hoped that our opportunities for observation might furnish us with the means of contributing somewhat towards the settlement of the most important of the questions discussed in them,—that of the identity, or the diversity, of the origin of the forms of disease called *variola* and *varicella;* but, though we have been almost wholly disappointed in that hope, we cannot, with propriety, defer still longer presenting a Review of them to the readers of this Journal. Varioloid diseases have, indeed, become comparatively rare in London during the last few months, and we have been led to believe that so populous and extensive a city as our metropolis, is not a favourable place for the investigation of the question above stated: the density and great extent of the population; the daily mutual intercourse of persons residing in various remote parts of the town; and the want of exclusive medical practice by any individual in any particular district, rendering it extremely difficult, if not impossible, to determine in a satisfactory manner the source of either of the forms of diseases above mentioned, in the generality of cases at least, if not in every instance, when communicated by *contagion;* (which is, perhaps, the only means of communication that, in the present state of our knowledge, can furnish valid and positive evidence for the decision of the question:) for, though we may ascertain that a person having the form of disease termed varicella, (for example,) had been exposed to the contagious influence of small-pox at such a period as would answer to the production of the disease, according to its ordinary process, we cannot, for the reasons above enumerated, feel assured that this person had not also been exposed to the contagious influence of chicken-pox at a similar period. The closest investigations of this kind with which we have become acquainted, are those of Dr. Macleod, (*see his papers on the subject in the Numbers of this Journal for July and August last,*) and the results of them support, very forcibly, the proposition of the identity of origin of the two forms of disease in question; a proposition which is also rendered feasible by the results of his experiments of a more positive character, in the cases of Henry Oldfield, (*see page* 10 *of the Journal for July last,*) and the children inoculated with matter taken from the eruption in his disease. This question we state to be the most important of those discussed in the works we are about to take into consideration, because of its relation to the advantages of the practice of vaccination: for, should it be proved that *varicella, horn-pox, water-pox,* &c. are modified forms of *small-pox,* the arguments that have been advanced against that practice,—by bringing forward the instances of *small-pox* (as the opposers of vaccination have called those diseases, when of any severity of character,) which have appeared in individuals subsequently to cow-pox,—will lose all their force, because it will be proved that small-pox itself must have commonly recurred in the same individual, since those diseases have in all times been common in persons who have undergone the last-mentioned affection; and they have, also, not unfrequently appeared epidemically in as severe forms as those which they have lately assumed in persons who have experienced cow-pox only. We shall, in the first instance, give an exposition of the matters most intimately related to this question, and then notice the rest of the important parts of the works before us.

On observing the appearance and progress of the varioloid disease which was lately epidemic in Edinburgh, Dr. Thomson says that he had many opportunities of perceiving the uncertainty of the grounds upon which, in particular instances, a case was declared to be chicken-pox or small-pox. "It appeared to me," he adds, "that practitioners were in general very unwilling to suffer themselves to be shackled by an adherence to any definitions of nosologists, and that, in deciding on doubtful cases, a reference was usually made to some ideal standard, not only indefinite in its nature, but varying from time to time, and seeming to be influenced by moral as well as by medical considerations." He often had occasion to observe *natural small-pox*, *modified small-pox*, and what he had been accustomed to regard as *chicken-pox* co-existing in the same situations, and appearing in their progress to produce one another. The two forms of small-pox and chicken-pox were to be seen prevailing at the same time in every quarter of the town, and often in patients living under the same roof, and even in the same room. He soon began to suspect that the several forms of disease above enumerated had one common origin; and his chief difficulty in admitting this conjecture, arose, he says, from his respect for the authority of Drs. Dimsdale, Heberden, Willan, Mr. Moore, and other authors of eminence, whose opportunities for observation were, probably, greater than any he had enjoyed. On mentioning, however, his supposition to some of the older and more experienced practitioners of his acquaintance, he was surprised to find, that, "though their general impressions were at variance with it, yet they were unable to point out any precise facts, derived from their own experience, by which it was contradicted." He therefore determined to keep this question in view during his observations of the progress of the epidemic, and to be guided solely by the results of his experience. One of the inferences he has thence formed is, that variola, horn-pox, and varicella, have a common origin, and mutually give rise to each other.

In all the three different classes of persons who were affected with this epidemic, that is to say, 1°, those who had neither experienced small-pox nor cow-pox; 2°, those who had experienced small-pox, and not cow-pox; and, 3°, those who had experienced cow-pox, and not small-pox; the disease appeared to him to exhibit certain general and common symptoms, while in each class it possessed more or less of a particular character, the principal varieties of which, he considers, may be referred, "first, to differences in the degree and duration of the eruptive fever; secondly, to differences in the number, form, contents, maturation, and decline, of the pustules; and, thirdly, to differences in the occurrence, degree, and kind, of the secondary fever supervening in the severer cases of the disease."

*In persons who had neither passed through small-pox nor cow-pox*, the eruptive fever was in general severe. It usually continued three days before the appearance of the eruption, though the period of its existence varied from that of one to that of four days. It was not possible, in the opinion of Dr. Thomson, to foretel, either from the degree or duration of this fever, whether the eruption which was to follow would be "of a vesicular or pustular, of a mild or a malignant, of a distinct or a confluent, form." The eruption was almost always papular in its origin.

"In a small number of cases," he says, "in which the eruption has been scanty, the papulæ have become vesicular on the first or second day, have continued such nearly to their appearance, which has usually happened before the end of the fifth or sixth day, and have left behind them only a slight roughness, or small thin scales upon the skin. The cases to which I allude have occurred in situations in which confluent and malignant small-pox existed, and to the contagion of which they could be distinctly traced. Had it not been for this circumstance, I should never have had any doubt of these cases having been examples of genuine chicken-pox. This variety might be termed *mild vesicular* small-pox.

"In other instances, in which the papulæ have from the first appeared vesicular, the vesicles, after continuing pellucid for two or more days, have become filled with a whitish fluid, sometimes resembling milk and sometimes pus, which dried into small crusts or scabs. It was impossible, during the vesicular state of the disease in these cases, to say whether the vesicles would become pustules; whether, when they became pustules, they would continue prominent or become depressed in their centres; and whether they would decay by the sixth or by the ninth day. In this variety, though the disease might have been regarded as chicken-pox in its commencement, it was impossible, by any characters with which I am acquainted, to have distinguished it from small-pox in its termination. This variety may be termed *vesico-pustular* small-pox."

In the greater number, however, of cases of this class, the papulæ became pustular at any early period of the eruption. In most instances in which it assumed the form of *distinct* small-pox, "*the appearance of the greater part, or of the whole, of the eruption has been simultaneous;* but in several, particularly in children, *it has come out in successive crops:*" two phenomena which we shall have occasion, hereafter, to notice in a particular manner in regard to the proposed means of diagnosis between variola and varicella. In another variety, the eruption not unfrequently "assumed the form which has been denominated *horn-pock*, *stone-pock*, and *warty* small-pox."—"In very few cases of the distinct small-pox have any symptoms of secondary fever occurred; and, even when they have occurred, they have been but slight, and of short duration. In most of these cases, also, there has been but very little, if any, of the smell peculiar to small-pox.

"In a large proportion of cases in this epidemic, the small-pox have been of the coherent kind, a sort intermediate, as it were, between the perfectly distinct and the confluent, which might, according to the quantity of the eruption, and the mildness or malignity of the constitutional symptoms, be referred to the one or other of these species. This variety has usually been preceded by a smart attack of eruptive fever, in general abating on the appearance of the eruption, but in other instances continuing for some days after this has taken place. In a few instances, a second attack of this fever has been observed to occur about the third day of the eruption; in some cases followed by a fresh eruption, and in others without that effect being produced. Inflammation of the fauces has been a very common occurrence in this as well as in all the other varieties of the epidemic, sometimes accompanied, and always much aggravated, by the appearance of pustules in these parts."

The papulæ in the coherent variety were not unfrequently observed to be vesicular before becoming pustular. "The size of the pustules has varied from a pin's head to that of a horse-bean. Their figure has been regular and irregular, lenticular, conoidal, and globate; in some instances with, and in others without, *central depressions.*" We stop in our analytical course to remark, that Dr. Thomson has not made it evident whether he means that the pustules in some instances *had*, and in others *had not*, the central depression, in the same person or in different persons. We shall presently show that this circumstance has been made a matter of great importance with those who have pretended to establish a distinctive diagnosis between variola and varicella.

The rest of the description of this form of the epidemic comprises nothing that seems to be of peculiar importance in regard to the question we propose expressly to consider.

It appears that Dr. Thomson witnessed 205 cases of natural small-pox in persons of this class; and of these fifty died,—a rate of mortality affording (he considers) "an undoubted proof of the malignant nature of the epidemic in its unmodified state."

In *the varioloid disease in those who had previously passed through small-pox*, the eruptive fever in the greater proportion was severe, but in some cases so mild as scarcely to have been perceptible. Forty-one cases of this kind were seen by Dr. Thomson "in individuals who had distinctly gone through either the natural or inoculated small-pox, some of whom had even been deeply marked by this disease."—"In a considerable number, the eruption, in its appearance and duration, has resembled the affection which has usually been described as chicken-pox, both in its vesicular and pustular forms. In others, the eruption has resembled that of distinct small-pox; and, in others again, small-pox of the coherent kind."

We cannot follow Dr. Thomson through his details of the various appearances of the eruption; but we may add, that he says "it is remarkable how much the appearance of the eruption in this class resembled those which have been described by the older authors as *spurious* small-pox, under the names of chicken-pox, sheep-pox, swine-pox, wind-pox, siliquose-pox, bladder-pox, &c. &c." Dr. Thomson has not stated whether the pustules possessed the *depression in their centre*, or the *cellular structure*,—stated by some authors as diagnostics of small-pox,—in any case in patients of this class. "In one instance only," he says, "has the disease occurred for the third time; and what is singular, it was more severe in the last attack than in either of the two former." Details of this kind are extremely desirable: without them, we have no satisfactory evidence of the two prior affections having been small-pox, whilst a doubt remains respecting the diversity of the origin of the diseases under consideration.

The circumstances noticed in the following paragraph also require particular illustrations, in order that they may appear of much importance in our views of the nature and origin of the diseases under consideration.

"Besides the forty-one cases which I have myself seen, I have been informed of thirty others in which the present epidemic has attacked those who had previously passed through small-pox; and of this whole number seventy-one, three have died, giving a proportion of deaths nearly as one to twenty-three. It deserves to be remarked, that, in two of the instances in which the disease proved fatal, the eruption in the first attack, though it could be traced to the contagion of small-pox, was vesicular rather than pustular, and that in both the disease recurred a few weeks after the first attack."

*The varioloid disease in those who had undergone the process of vaccination*, was attended, in a great proportion of cases, with "severe" eruptive fever, of from one to four days' duration.

"The varioloid eruption itself has always first appeared in the form of papulæ, and many of these have decayed without becoming either vesicular or pustular. In a considerable number of cases, the papulæ, which were of a red colour, and felt perceptibly hard to the finger, were sometimes covered, in the course of a very few hours, with thin vesicles, containing a limpid fluid; in other instances, the vesicles were not formed till after a longer period. These vesicles have often decayed or burst by the third or fourth day, without becoming pustular, the fluid in them acquiring a slightly-turbid milky colour, and have generally left behind them only a tendency to desquamation or scurfiness of the cuticle. In this, as in the other varieties of the disease in the vaccinated, the vesicles have often been surrounded by a red areola, which seemed to be of greater or less extent and duration, according to the more or less inflammatory nature of the cutaneous texture in different individuals. This variety has, in the mildness of the eruptive fever, the strictly vesicular character, short duration and mode of disappearance of the eruption,—corresponded so exactly with the descriptions usually given of the mildest varieties of chicken-pox, as not to have been distinguishable from that affection.

"In other instances, the vesicles which have remained for one, two, or more days, filled with a limpid fluid, have in their progress acquired a milk-like or purulent appearance. In decaying, some of these have become depressed in their centres, and have formed thin scabs, which have begun to separate by the fourth, fifth, or sixth day of the eruption; and in other instances not before the ninth, or even twelfth, day. In some instances the vesications continued limpid from three to five days, then became pustular, and remained in this state for two or three days longer before they began to be formed into crusts or scabs. In the greater number of these cases the crusts and scabs have fallen off without leaving behind them any tubercles, blains, or pits. In a few instances, however, the crusts and scabs have not fallen off before the twelfth, and sometimes not before the seventeenth, or even the twentieth, day; and in these cases tubercles, blains, and pits, have usually been perceptible at a much later period of the eruption."

In a very considerable number of cases, Dr. Thomson adds, the varioloid disease, whether it occurred in a vesicular or pustular form, "assumed in its progress the appearance of distinct small-pox. It has appeared to differ chiefly from that disease in the small size of the pustules, in their containing often a milk-like rather than a purulent fluid, and in their beginning often to dry and scab by the fourth or fifth day. It has not been possible, from the appearance of the eruption when vesicular, to say whether it would continue such, or become pustular; and of the vesicles becoming pustules, *comparatively few have exhibited any depression in their centres.*" In "a small proportion," the eruption assumed the appearance of what has already been described under the name of *coherent* small-pox. In all the varieties, the eruption almost always came out in successive crops, fresh eruptions sometimes appearing even after the fifth day. Of the 310 individuals, seen by Dr. Thomson, who were affected with this epidemic "after having gone through the process of vaccination," only one died. In above forty of those who had been previously vaccinated, "the varioloid disease occurred for the second time, after intervals varying from a few days to several years. In some of these cases it exhibited, in the first attack, the appearance of chicken-pox, and in the second that of small pox; in others, again, in the first attack it resembled small-pox, and in the second chicken-pox. In some, the disease has in both attacks resembled chicken-pox, and in others small-pox." The foregoing are the principal points that seem most worthy of citation respecting this class of patients. The application of them will be presently shown.

It must be remarked by every reader, that the above descriptions involve in so blended a manner the characters both of chicken-pox and small-pox, that it follows, either that the two diseases must have been distributed throughout the city in an equally general manner, or that the various forms of the disorder have one common origin, and are modified by diverse circumstances of locality and of the constitution of the subjects of them. We shall now consider the arguments that present themselves, respectively, in support of the two different views of this subject above designated.

Mr. Bryce, Dr. Abercrombie, and Dr. Alison, all of whom have had extensive opportunities for witnessing the epidemic disease under consideration, maintain the opinion, most generally prevalent, of the diversity of the two diseases, and they state that there are signs by which they may be distinguished. Mr. Bryce says that chicken-pox, as he has observed it,

"Generally attacks with little or no fever, the appearance of vesicles on the shoulders, neck, and breast, being often the first symptom observed. The vesicles are often, when first seen, about the size of a split-pea, perfectly transparent, and covered only by the cuticle, as thin as that separated by a scald or by a blister: they generally have at first an inflamed areola, but this seems also to be confined to the cuticle, and there seems to be little, if any, hardness in the true skin beneath or around them. On puncturing the vesicle, the clear lymph is wholly evacuated, the cuticle falls flat down, and very little, if any, hardness is perceived on passing the finger over the collapsed vesicles. The vesicles generally increase in number for several days; and, while new vesicles are appearing on some parts of the body, those which had first come out are beginning to shrivel, and the fluid contained in them has become somewhat milky. Many of them are broken by the second or third day, and have a small crust formed on the cuticle, which adheres to the skin beneath, and is surrounded by an opaque or milky fluid, confined by the shrivelled cuticle. When the eruption is numerous, the body has the appearance of having been exposed to a shower of boiling water, each drop of which had occasioned a vesicle or blister; and these are generally, on the second and third day, when turgid, broader at the summit than at the base. When the vesicles remain unbroken for four or five days, as is sometimes the case, the covering of cuticle, as well as the contained fluid, become opaque, and the latter purulent. The vesicle is then much flattened; and in this stage of the disease it is scarcely to be distinguished from small-pox, unless by the very thin, delicate, and shrivelled appearance of the covering cuticle.

"The most characteristic symptoms of this eruptive disease appear to me to be the slight degree of eruptive fever, the rapidity with which the fluid is secreted, or rather effused, into the vesicles, and the perfect transparency and thinness of the whole vesicles, even in the unvaccinated, during the first and second days of their appearance, very unlike the firm, hard, and solid tumors which form the eruption of small-pox at the same period of that disease."

Dr. ABERCROMBIE, describing chicken-pox, says

"This eruption is preceded for a day or two by fever, generally slight. When a single specimen of the eruption is minutely examined, it is found to be from the earliest period a watery vesicle, covered by a thin pellicle of skin, which usually has a loose shrivelled appearance. The vesicles increase in size for three or four days, and then generally burst; the fluid drying into loose scaly crusts, of a light-yellowish colour. In some constitutions, and on some parts of the body where the cuticle is unusually strong, the vesicles may continue unbroken for a longer period, perhaps to the sixth or seventh day, and in these cases they assume a yellow puriform appearance, considerably resembling the pustules of small-pox. If one of the vesicles of this eruption be punctured on the second or third day, so as carefully to discharge all the fluid, the pellicle which covered it falls down; and, the finger being then carried over it, it is found to be correctly on a level with the surrounding integuments."

Dr. ALISON states, as the diagnostics of chicken-pox,

"1st. The slight degree of eruptive fever. 2d. The sudden formation of the vesicles. 3d. The greater dimension of the vesicles at the summit than at the base. 4th. The want, in these vesicles, of central depressions. 5th. The want of hardness in the base of the vesicles. 6th. The falling down of the vesicles on the letting out of their contents. 7th. The uniformity in the appearances of the eruption in the vaccinated and in the unvaccinated; and, 8th. The difference which exists, during the first three or four days of the disease, between it and modified small-pox. The fourth and the eighth of these characters are the only grounds of distinction between this new species of chicken-pox and modified small-pox, which I have not already considered."

Mr. MOORE describes the characteristics of the eruption in the same disease in the following manner. On the first day, a small vesicle may usually be seen, and little or no coagulable lymph is effused to thicken the cuticular membranes; a serous secretion, the production of cutaneous inflammation, is poured out under the *rete mucosum* almost with the rapidity of a blister, and little vesicles are quickly formed, more pellucid than those in small-pox, yet surrounded with inflamed bases of various breadths. On the second day, the vesicles are larger, but neither concave, clouded, nor hedged round with coagulable lymph. But, as they fill, the fluid separates the rete mucosum and cuticle from the cutis; and, as one side yields more than another, the vesicles occasionally become oval, or lenticular, or in some degree irregular. Often, however, they retain a regular round figure, but not so constantly as in small-pox. But, he adds that, when the disease is violent, the inflammation is active; the vesicles run into suppuration, are enlarged and protracted in duration, obscuring all the discriminating marks.

The incongruities in those descriptions, and in respect to the chicken-pox of HEBERDEN, were so well pointed out by Dr. MACLEOD, in his papers in a late Number of this Journal, that we need not stop to consider them here. We proceed, therefore, to the observations of Dr. HEIM, of Berlin, who has paid much attention to this subject, and who has had ample opportunities for observation. He says that varicella is met with in three forms:

"1. Water-pox. The pocks contain a white transparent fluid, but no true pus; they are of different sizes, and have a slight indentation in the middle. If the lymph should assume the purulent colour, the pocks will look exactly as if they were variolous. 2. Horn-pox. These appear as pointed elevations, without any indentation; are hard or even warty, and do not contain lymph. 3. Swine-pox. These are mostly of an oblong shape, sometimes quite round. They become much larger than variolons pustules, have occasionally a red circle at their basis, suppurate, and are fully distended with their contents, leaving irritable ulcers and cicatrices."*

Dr. Heim enumerates, in a parallel, several diagnostics of small-pox and varicella; but the greater part of them are liable to the objectionable remark of Dr. Macleod,—that, when men "meet with an eruption which is too mild in itself, or too short in its duration, to come up to their idea of small-pox, they satisfy themselves that it must necessarily be chicken-pox;" for the greater proportion of his distinctive marks may, with much plausibility, be referred to a more or less intense form of the same disease. Before we notice this subject more minutely, we should, however, adduce an account of what have been proposed as the characteristics of small-pox, to place in comparison with those above asserted to appertain properly to varicella. We shall take this account from the work of Mr. CROSS; as this author has availed himself of the latest, as well as the more early, observations that have been made on this subject in different parts of Europe.

Small-pox, Mr. Cross says, "has been generally found to be preceded by three or four days of severe indisposition, with fever, vomiting, pain in the back and limbs, and tenderness in the epigastric region:" but he acknowledges that the preceding indisposition "can never be considered decisive of an eruption being variolous, if the subsequent progress of the disease, or some collateral circumstance, does not explain it to be so."—"The coming out of the eruption has also been regarded as characteristic, appearing first upon the face, and being fully

* We take this extract from the translation of Dr. HEIM's Memoir on this subject given by Mr. Cross.

completed in four days." These are, we think, but doubtful diagnostics; and SELLE and some other authors speak of well-marked small-pox appearing, as varicella is said to do,—first on the body and extremities, and lastly in the face. The period of maturation, Mr. Cross acknowledges, presents no certain indications of small-pox. The crimson areola round the pocks, the fetor, and the secondary fever, may be attributed, with plausibility, by those who adopt the opinion of the identity of the origin of the two forms of disease, to an accidental diversity in the degree of local irritation and fever. The same remarks are, perhaps, equally well applicable to the presence of a slough at the bottom of the pocks, which JOHN HUNTER attributed to the *peculiar kind of inflammation* which he supposed to be present in varicella. This diagnostic was also admitted by Dr. ADAMS. (See vol. xii. p. 7, of this Journal.) Mr. Cross considers the existence of this slough as "the most direct rule that has been given;" but, he adds, it "deserves not to be implicitly relied upon, its presence in all the pocks only proving small-pox, but its absence from a great proportion of them not proving the contrary. If a pock does not suppurate, will the slough ever be formed? if it will not, then the suppuration is as much the test as the slough itself." The appearances of the scars furnish some diagnostic marks, according to Dr. Cross and some other estimable authors. Dr. Heim says,

"There is no doubt that we can distinguish the scars left by chicken-pox from those left by small-pox, and thus be enabled to tell, after a number of years, whether a person has had one or the other; which is of great importance in judging of the protecting power of cow-pox. This however depends upon the scars being complete and distinct, which we can only expect to be the case when the pustules have gone through their proper course, in which they may be interrupted by being bruised or scratched, and probably also by a scrofulous or venereal state of the system. So likewise the pustules of chicken-pox may degenerate into an ulcer; and this is the case when fluid is formed in them after the twelfth day, and no regular scab is formed. The varicellous scars, under these circumstances, lose more or less of the peculiarity which distinguishes them from variolous scars. The diagnostic signs are as follows:

"1. The basis of the scars from chicken-pox is whiter than the skin of the rest of the body, and smooth as the shell of an egg; that of variola is not at all whiter than the skin, and is uneven as the surface of a lemon. Besides, the former shows no points or indentations, which are not wanting in the scars from small-pox, in which we find two or three dark points, and always more the larger the scar. In the varicellous scar we never find hairs in the hairy parts, as the eyebrows, scalp, chin: the reverse is the case in the small-pox, two or three hairs being often found upon the surface of one scar.

"2. The varicellous scar is rounded and smooth in the circumference, possesses at its border the colour of the surrounding skin, and forms at the base a hardly-perceptible depression, so as even to assume a convex appearance. Some old scars in adults are wrinkled at the circumference, and even at the basis; but if they are stretched, the wrinkles disappear. The circumference of the variolous scar, on the contrary, is always more or less notched, is not different from the common colour of the skin, and, however strongly stretched and extended, never becomes perfectly even and smooth, either at its basis or its circumference. When stretched, it looks as if fine lines were drawn across it.

"3. The shape of the varicellous scars is generally round, sometimes oval, and very seldom irregular. When they have angles, it is when two are joined together, from two pocks which were approximated to each other, or which ran into one another. The shape of the variolous scar is seldom round and oval, often notched and forming all sorts of angles.

"4. The depth of the varicellous scars varies much, partly from their age, and partly from the situation which they occupy. In the neck and face, particularly on the forehead, they are deepest; much less deep upon the extremities, back, and abdomen. In very old scars all depth is lost, so that they are exactly level with the surrounding skin, or even become elevated a little above it, particularly on the back and abdomen; but they remain unaltered as to their colour. In variolous scars the depth always diminishes by time, and often entirely disappears without the least trace of them being left behind.

"5. The varicellous pocks never leave many scars, often not above twenty, sometimes only one or two, and these happen most commonly on the face, above or on the nose, on the forehead, &c. More rarely they are found on the abdomen, back, or extremities, but always in greater number. The variolous pocks often leave many, even innumerable scars, mostly upon the face and hands; those upon the abdomen and back entirely disappearing with time.

"6. The scars from accidental injury to the cutis, as from small ulcers, pemphigus, erysipelas, zona, the removal of warts or moles from the skin with caustic, from the bite of leeches, the use of the tartarized antimonial ointment, (all of which may sometimes possess a resemblance to the scars left by pocks,) are distinguished from them by possessing a hardness to the touch, which is never the case in the scars left by varicellous pocks, these being always very soft to the feeling."

Mr. Cross considers that the *structure* of the variolous and of the varicellous pocks differs in a remarkable manner. Each pock in varicella, he says, is formed of only a simple vesicle; whilst the pock of variola is composed of several cells, filled, by the fourth day of the eruption, with a limpid fluid. "To a cellular structure of this sort, (he says,) the term *pock* should perhaps be strictly confined. The walls of the cells being perfectly transparent, the disposition of them during life is not easily ascertained; but they are more readily examined in the dead subject, and the arrangement of the partitions, first described to me by Professor MACARTNEY of Dublin, (whose researches in minute and comparative anatomy have been so justly appreciated,) I have found to answer to the axis, spokes, and circumference of a wheel. Besides the cutis, which is external, and the very vascular surface of the skin forming the basis of the pock, there is the cellular structure formed by the rete mucosum,* or by a freshly-organized substance thrown out by the cutis itself, and excavated into cells for holding the fluid of the pock. The walls of these cells secrete the contained fluid, and, if this be partly let out by a puncture, the drying of the lymph closes the point opened, and the fluid is again secreted, distending the pock to its former shape. Inoculation is practised most successfully with the transparent lymph taken from a pock in this stage of the eruption."

This cellular structure, with the scar peculiar to it, and the central depression in the pocks, are, it would appear, the only precise marks of diversity between variolous and varicellous pocks. The whole of the rest may, without any forced reasoning, be attributed to diversities in the degree of intensity of the disease; and Mr. Cross acknowledges that "mild cases may occur which cannot by any short rule be determined small-pox," because in such cases all the circumstances said to be characteristic of small-pox may be absent. The appearing of the eruption in *successive crops*, which has been supposed a diagnostic of chicken-pox, has been observed by Dr. Thomson as an "extremely common occurrence in undoubted cases of modified small-pox; not unfrequently in secondary small-pox; and even sometimes, though less frequently, in cases admitted to be natural small-pox. The *odour* said to be peculiar to natural small-pox, has been perceived by HUFELAND (*Bemerk. ueber die Naturlichen und Geimpften Blattern*, p. 54,) and VOGEL (*Handbuch der Praktischen Arzneywissenschaft*, p. 178,) in the first stage of what they considered to be well-marked chicken-pox. The whole of the circumstances stated by Dr. ALISON as diagnostics of chicken-pox, excepting the fourth and eighth, have been negatived by Dr. THOMSON; and the latter, Dr. T.

---

* The vaccine pock bears a strong resemblance to the variolous; yet, from the foveolous appearance of the scar, it seems probable that the cells are differently arranged. Dr. WILLAN (on *Vac. Inoc.* p. 9,) speaking of the cells of the vaccine pock, says, "these are, perhaps, only a portion of the cellular membrane distended by the effusion of lymph." This observation shows how little attention he gave at the time to the situation and structure of the vaccine-pock; the cellular substance being deep and out of the way, with the dense and less vascular part of the skin between it and the basis of the pock. COTUNNIUS (*De sede Variolarum.* p. 202,) came nearer the true situation of a pock, placing it neither in the cutis nor cuticle, but rather between the two.

remarks, is one of these vague statements which he has not found any one able to reduce to any precise ideas. The anatomical structure of the pock is the only point left to rest a precise distinction on; and it is not, we think, very good logic, to found a diagnostic on a phenomenon which is frequently not present. If the disease may run its course without the appearance of a depression in the centre of the pocks, and if the cellular structure is also occasionally absent, it follows that these are circumstances accidental to the proper phenomena of the disease, and therefore cannot, by sound reasoning, be regarded as its characteristics. The establishing them as such, is a *petitio principii;* similar to that by which *woolly hair* has been said to be the characteristic of a distinct primitive race of men,—an assertion which appeared plausible until it was found that these woolly-haired men have descended from a race which had not woolly hair. In the want, then, of decisive *physical* evidence by which the question under consideration may be settled, we are led to enquire what light may be thrown on the subject by *moral* considerations; and these, as Dr. Thomson and Dr. Macleod have shown, tend very forcibly to support the opinion of the identity of the two diseases, by the strong presumption, derived from close investigation and correct reasoning, that what is called variola has given rise to varicella, and varicella to variola.

The cases of the Oldfields (page 10 of the Journal for July), and the Hutchins', (page 96, August), related by Dr. Macleod, are closely in point in this respect; and Dr. Thomson mentions many others which contribute to the support of the same opinion. He notices, particularly, a case which was regarded as chicken-pox by Dr. Alison. A child had died of confluent small-pox, a few days before this, in a house on the opposite side of the street to that in which the subject of this case lived, and "there was at this very time another child recovering from a severe case of coherent small-pox, in the floor above that where the child having the vesicular eruption lived. The grandmother of this child, a determined enemy to vaccination, seemed much pleased to think that her little charge had got the small-pox in so mild a manner, while around her even the children who had been vaccinated were seized with this disease, many of them in a much severer form. It was with some difficulty that I could get this woman to confess that she had not only gone herself, but that she had repeatedly carried her infant charge into the room where the child I have mentioned was recovering from the small-pox; and, by way of excuse, she alleged that she had never remained there longer than a quarter of an hour at a time."

We ourselves witnessed a case of the simplest and mildest vesicular disease, (in which the vesicles were formed rapidly—in a few hours; came out in successive crops; were in the highest degree pellucid; formed more than half a sphere; were broader at the summit than at the base; had no depression in the centre in any one of them; and disappeared on the third day after their full formation, without having become at all pustular, leaving the cuticle in a shrivelled state,) in an infant eight months old, which had passed through neither small-pox nor cow-pox, that appeared a week after the death of a child in the same house, on the floor below it, from confluent natural small-pox, and a few days after three children of the same family had small-pox break out;—in two of whom the disease assumed the most severe petechial and confluent form, and terminated fatally. The infant in question died a few days after the disappearance of the eruption, apparently from inflammation of the lungs, signs of which were evident before the eruption broke out. This child had not been taken into the streets for some weeks previously, and we could not discover that chicken-pox existed in the neighbourhood.

On the other hand, there are equally good grounds for the presumption that chicken-pox has given rise to small-pox. The work of Dr. Thompson presents abundance of evidence on this point, and both the propositions just mentioned have been supported by the observations of Dr. Bent, (*see his Paper in the Number of this Journal for December* 1818,) and by some other authors, whose works have been already noticed in former Numbers of this Journal. After referring to these observations in a particular manner, Dr. Thomson says,

"It was indeed possible to conceive that the contagion of chicken-pox might, in any one of the situations to which I have referred, have co-existed with that of small-pox; but that it should have co-existed in them all with the contagion of small-pox, and that it should have operated just at the time the infection of small-pox ought to have taken place, appeared to me to be suppositions altogether gratuitous, and so totally devoid of probability, as to render it impossible to entertain them even for a moment."

Dr. Abercrombie, however, in opposition to the statements just adduced, says, "I state with confidence that I have often seen small-pox epidemic, without having observed mixed with the cases of it any examples of the vesicular disease of Mr. Bryce: nor have I ever been able to trace an example of the latter to the contagion of small-pox, always keeping in mind the important distinction betwixt this vesicular disease and the horn-pock, or modified small-pox, which has been very generally confounded with it."

Heim, as we have already shown, regards *horn-pox* itself as a variety of varicella; and, from the short description which he has given of the disease to which he applies the term *horn-pox*, it appears that he meant to designate the same form of disease as that to which the same term has commonly been applied amongst us. Dr. Abercrombie, however, considers horn-pox as small-pox modified either by previous small-pox or by cow-pox. On this point Dr. Thomson remarks, that

"If by horn-pox we are to understand that form of the varioloid disease in which the contents of the vesicles and pustules are converted into brown-coloured, semi-transparent, horn-like, scabs, which, in falling off, usually leave behind them slight temporary elevations of the skin in the spots which they occupied; then I am prepared to state, that, in the course of the present epidemic, horn-pox have occurred to my observation much more frequently in natural small-pox than in small-pox modified by vaccination, and therefore that I cannot by any means admit the horny character of the scabs in varioloid diseases as affording any diagnostic mark, either between modified small-pox and chicken-pox, or between modified small-pox and natural small-pox."

The circumstance which seems to militate most strongly against the notion of the identity of variola and varicella, is that the form of varicella does not seem to have been ever known to have arisen from inoculated small-pox; for, had such an occurrence happened, it could not have failed to strike practitioners in a forcible manner, and, of course, to have led, long since, to the opinion of the identity of the origin of the two forms of disease:—we mean, to the general adoption of such an opinion; for it has at all times, since small-pox has been known in Europe, been entertained by men of eminence; as MEHRBECK, in particular, has shown in his very erudite dissertation on this subject. As the disease from inoculation is generally milder than that from contagion, it would seem that varicella would be more likely to arise from the former than the latter means of communication, were they of common origin. Another argument against the same opinion is, that fatal cases of small-pox have occurred, in a few instances, within a few weeks after chicken-pox, and severe cases, not unfrequently, at a more distant period; whilst it is generally acknowledged that secondary attacks of eruptive diseases are almost universally milder than the primary. Some change may be supposed to have taken place in the constitution of the patient in the interval of the attacks, by which the latter may have been rendered so much more severe than the former; but such a supposition is not a very plausible one. Were the two diseases specifically identical, we should expect to find the first attack assume the form of small-pox, and the secondary, or subsequent, attacks, that of chicken-pox, in the generality of cases at least, if not in almost every instance: but this is not the case; the instances of small-pox succeeding chicken-pox, are by no means of rare occurrence.

It might, on first consideration, be supposed that it were possible to settle this important question by an experiment, readily practicable,—that of inoculating a person liable to the variolous disease with varicellous matter. This experiment has been tried, but the results are not conclusive, for many reasons. Without adverting particularly to the experiments of some of the older authors,—who believed, as well as HEBERDEN and DIMSDALE, (who are said by our modern writers not to have known how to distinguish small-pox and chicken-pox,) that varicella might be communicated by inoculation, and who speak of its having been done, and of a disease thence arising which might be mistaken for small-pox,—let us notice only those of some modern physicians, that have been instituted with a regard to the question under consideration. By Dr. Alison's letter to Dr. Thomson, it appears that Mr. M'INTOSH inoculated *one* child with *lymph*, and *two* others with *pus*, taken from a child having what he (Dr. A.) considered to be chicken-pox. "The first produced no effect; the two last produced a good deal of local erythematic inflammation, with vesicles on the inflamed portion of the skin, but no general eruption." The results of this experiment cannot be allowed to have much weight, because the eruption appeared on the child from which the matter was taken, "on the third or fourth day after *vaccination*," and was therefore not a fair subject for the enquiry. Different results were witnessed in those related by WILLAN: he says, that two children were inoculated, in 1798, with matter taken from their brother, who was affected with chicken-pox. In one of these children, the punctures of the inoculation inflamed, and became, by the seventh day, equal in size to that of a variolous pustule. In the other child, the redness surrounding the puncture gradually disappeared, leaving no mark whatever behind; yet in each of these children there appeared, on the thirteenth day after inoculation, an eruption of vesicles, which went off in three days, leaving the children free from indisposition. Although these cases tend somewhat to show that varicella may be communicated by inoculation, they do not furnish any satisfactory grounds for precise inferences on this point: it may be considered doubtful whether the eruption arose from the inoculation, or from infection from their brother, or from the same source as he derived his disease. If it be asked of those who adopt the opinion of the identity of origin of variola and varicella, why small-pox was not produced by the inoculation, if any disease was produced by it, it may be replied, that chicken-pox is a form of small-pox, in which the *specific virus* is not well developed, from the want of sufficiently intense inflammatory action of the vessels by which the fluid of the pocks has been formed;—an argument advanced by Dr. Macleod in his papers on this subject, and illustrated by him by a case in point. It seems worthy of remark, too, that many persons have been known to resist small-pox by inoculation, who have afterwards taken it by infection. Dr. Thomson says, that this was noticed in a "considerable number" of instances, during the existence of the epidemic at Edinburgh. It is not unreasonable to suppose, then, that small-pox may arise from chicken-pox by infection, though it cannot be communicated by inoculation; or, in other words, that a more severe form of disease may be communicated by infection from chicken-pox than by inoculation; as we commonly observe in the unequivocal form of variola.

Mr. Bryce says, that he has taken matter from the vesicles of chicken-pox, and has known it taken from them by others, "on five different individuals with the greatest care, at all periods of the disease, and at all seasons of the year; and have inoculated, and seen others inoculate with it, children who had never undergone either the cow-pox or the small-pox, to the number of thirteen different persons; yet in none of them was this disease, nor any thing like small-pox, ever produced." Were results such as those mentioned by Mr. Bryce, found to have been universal in an extensive series of trials, they would still furnish only negative evidence in respect to the proposition of the diversity of the two diseases: but even this kind of evidence in favour of it is not well established; for Dr. Thomson says, that he knows that experiments similar to those of Mr. Bryce have been made by different practitioners, in other places, with the matter of varioloid eruptions, conceived to be chicken-pox, occurring after vaccination, and after previous infection with small-pox; and though in general with the same results, yet sometimes with the effect of producing constitutional eruptions. Every reader must wish that Dr. Thomson had been more particular in his statements,—by giving the precise histories of some of those cases,—because general assertions of this kind will not satisfy the members of the profession generally, however highly they may estimate the talents of the author for accuracy of observation.

After describing several varieties of varioloid disease, affecting persons who had passed through cow-pox chiefly, which Mr. Cross considers to have all arisen from the contagion of small-pox, the author just mentioned says, "the cases which never advanced so far as to present indented pocks, I have not known submitted to any experiment; but the mildest examples, where such indentation existed, have been found communicable by inoculation, producing in a few instances small-pox in those who had not before had either that disease or cow-pox, whilst, in a small proportion of the vaccinated, they produced an eruption similar to themselves in mildness and duration." We make this extract merely to show that it is not sufficient that matter be taken from vesicles, in order that the disease may be produced by inoculation in other individuals; but that it is requisite that *that* matter be the result of a certain intensity of the specific action present. For, it appears that when the eruption consisted of pocks formed by *simple* vesicles only, —that is, probably, when the irritation of the cutis was but slight, and did not penetrate deeply into the cellular texture of the skin,—no disease was produced by it, by inoculation; whilst an eruption, considered by the historian of the cases to have been of the same origin, and affecting the same class of persons, in which the diseased action was more intense, and the pocks depressed in the centre or cellular, furnished matter capable of exciting the specific disorder. These facts are, we think, qualified to refute the inferences that have been drawn respecting the peculiarity of chicken-pox as a morbid affection, because it cannot be communicated by inoculation. It would appear, too, though Mr. Cross has not expressed himself quite clearly on this point, that the mildness of the disease produced by inoculation in the cases just alluded to, ordinarily bore some degree of direct ratio to the mildness of the source whence it was derived: a circumstance worthy of being remarked in respect to the results of the experiments related by Dr. Willan; in which chicken-pox, not small-pox, was, as it seems most probable, produced by inoculation with the matter of varicella.

We have noticed, we believe, all the most essential facts relating to this question that have hitherto been observed, excepting the results of the experiment of Dr. Macleod with the matter of the eruption of Henry Oldfield, (*see the Journal for July*, p. 10.) where small-pox was produced by inoculation from what Dr. Macleod says "would very lately have passed unquestioned as a case of chicken-pox." We did not ourselves see Henry Oldfield until after the eruption had disappeared; but we can add our testimony to that of Dr. Macleod as to the nature of the disease produced in the two children inoculated from him,—that it was well-marked small-pox.

Here, then, we terminate the discussion of this question, without coming to any other conclusion than that the inference which states the identity of the specific nature of chicken-pox and small-pox, is a more probable inference than that which asserts the specific diversity of the two diseases. There is much, apparently forcible, presumptive evidence in favour of the former proposition; whilst that for the latter is merely negative, and the arguments for it are all *ad ignorantiam*. The former is supported by the strong presumptive physical evidence which we have noticed, that small-pox has given origin to chicken-pox, and *vice versa;* and by the moral considerations, noticed by Dr. Thomson, derived from the fact that the two forms of the disease in question have been found to co-exist constantly in the same situations.

We shall now notice in a more particular manner than we have done hitherto the work of Mr. Cross. It is divided into two parts, and these again into several chapters, each of which comprises a particular view of the general subject of the work. The first chapter presents an account of the small-pox as it appeared in Norwich in the year 1819, and contains a very good description of the grand traits of the epidemy; but which our limits will not permit us to detail: we may, however, remark, by the way, that it appears to have possessed much severity of character; and the apathy of the lower classes of the people had left a great proportion of the population of the city susceptible of its full influence. But a very small number of these persons had passed through cow-pox. The number of deaths from small-pox in 1819, was five hundred and thirty; and about three thousand appear to have been affected with the disease, which is about a thirteenth part of the whole of the inhabitants. Small-pox had been unknown in the city, as far as Mr. Cross could learn, (excepting in one instance of a servant travelling that way,) for four years previously to the breaking out of this epidemy, which seemed to have been introduced by a girl, who, travelling thither from York, was exposed to small-pox at a market-town in the course of her journey, and had the disease appear on her arrival at Norwich.

The second chapter treats of the cow-pox, as far as regards the most remarkable circumstances relative to it which the author has witnessed, and the means taken to extend vaccination amongst the people. We find here the same apathy amongst the poor as is observed elsewhere: as soon as the ravages of the small-pox had ceased, the applications for vaccination ceased also, although a reward was given to the parents who brought children to Mr. Cross, (who was appointed to vaccinate the poor by a committee,) for this purpose. In September, 1819, only fourteen applied; but four in October, two in November, and none in December. It appeared, from Mr. Cross's observations, that about one person in fifty (supposed to be liable to its influence,) resisted inoculation with cow-pox; a proportion, he remarks, nearly equal to that of persons who resist small-pox inoculation. He inoculated one child six times, on each occasion making four punctures, with lymph immediately taken from a perfect vaccine vesicle, before the infection took place, and it then happened in only one of the punctures.

Although we cannot give a regular analysis of this chapter, we shall extract a few observations from it, which will be generally interesting, and which will, at the same time, show the accuracy with which the author conducted his investigations. One of his objects was to ascertain precisely the proportion in which persons having had cow-pox will, in general, become affected with small-pox contagion, and that in which cases of secondary small-pox will occur. With this view, he examined particularly, in one instance, the state of these circumstances in a certain number of families. In 112 families, comprising 603 persons, 200 had small-pox; one of whom had, three months previously, gone through chicken-pox, and another had the same disease a few weeks after the small-pox; ninety-one had been vaccinated, two of whom had modified small-pox, and one had chicken-pox; and the remaining 312 who had small-pox formerly, or possessed a peculiarity of system enabling them to resist it, had no eruptive disease of any sort during the existence of the epidemy. The ninety-one vaccinated persons were continually in the same room, and often lay in the same bed, with the variolous patients. Thirty-four were vaccinated during the epidemy, after the small-pox had appeared in their families, and were thereby prevented from having the latter disease. The remaining fifty-seven had the cow-pox at the following periods previously:

| | | | |
|---|---|---|---|
| From | three to five years | 3 | 57 |
| — | five to ten years | 37 | |
| — | ten to fifteen years | 11 | |
| — | fifteen to twenty years | 6 | |

Their arms presented the following appearances:

| | | |
|---|---|---|
| A small shining scar upon one arm | 3 | 57 |
| A small foveolous scar upon one arm | 8 | |
| A large foveolous scar upon one arm | 21 | |
| A scar upon each arm, in some indented or foveolous, in others shining like a cicatrix | 18 | |
| Two small foveolous scars upon each arm | 1 | |
| A scar scarcely perceptible | 6 | |

The two instances of modified small-pox occurred in those who had only one small foveolous scar.

The third chapter is on "small-pox and cow-pox occurring together in the same individual;" and contains some very interesting original observations, as well as references to the recorded facts and opinions, respecting this subject. Mr. Cross's observations tend to confirm those of Drs. Willan, Woodville, and Adams, on the period at which the cow-pox renders the system proof against the variolous poison by inoculation,—that it is effected by the eighth day. "The period at which small-pox," he adds, "in consequence of previous exposure to the contagion, may come out during the progress of the vaccine disease, is not so clearly ascertained, being a matter of accidental observation rather than of direct experiment. A few scattered and unimportant pocks may, under these circumstances, come out at the conclusion of the vaccine disease; but most writers in Britain seem to regard the fifth or sixth day from the vaccination as the latest time at which small-pox may appear, and go through a severe course in conjunction with the cow-pox."

His observations on the influence of small-pox on the progress of the vaccine vesicle, are peculiar to himself: he says,

"Although the eruption of small-pox may come out during the progress of cow-pox, the contrary does not take place; the vaccine vesicle never coming forward after the variolous eruption has begun to appear. Where small-pox has come out on the second or third day after the insertion of the vaccine virus, the latter has rarely produced any effect. After the vaccine pock has begun to show itself, I have met with small-pox coming out at all periods until near the termination of the vaccine disease, and I have convinced myself that this has greatly mitigated the violence of the natural contagion, because, of about thirty cases in which the two diseases have thus occurred together, none have died, and two or three only were apprehended to be in any danger. In general, the later the period at which the small-pox has appeared the more mild has it been rendered, and the more perfectly have the characters of the vaccine disease been maintained. The influence of the two diseases has been reciprocal. If the small-pox has appeared before the time when the areola should begin to form around the vaccine pock, the latter has subsequently been altered in its character, losing its circular shape, becoming irregular, flatter, sometimes having its contents nearly absorbed, leaving an empty bag, and ending in an imperfect thin scab; whilst the variolous eruption has proceeded in its regular course. But, where the appearance of the variolous eruption has been deferred until the areola had formed around the vaccine pock, this has ended in a regular scab, and the variola has generally, though not uniformly, proved mild; and often been confined to a few pocks, having sometimes the indented character, and drying up in a few days without maturating. Appearing later than the ninth or tenth day from the vaccination, they have always, according to my experience, assumed this mild form, been in small number, and lasted only three or four days."

Several cases, in which the progress of the two affections was somewhat different from that indicated in the above paragraph, are related in detail; and the chapter concludes with some general cautionary remarks, deduced from the circumstances considered in it. The chief of these are—

"Firstly, to vaccinate although the variolous contagion may already have been caught, as the small-pox, if not prevented, is most likely to be mitigated, should the vaccination take effect. Secondly, to avoid all unnecessary exposure of patients undergoing the cow-pox, to the variolous contagion, until the areola is fading; because, although a great proportion will escape, there is a chance of some being affected by it, and it is desirable to shun even the most mitigated form of the variolous eruption. Thirdly, when small-pox is prevailing, to give no decisive opinion of protection by cow-pox until the scab is about to fall off, that parents may not be deceived, nor the powerful means of safety be unjustly brought into discredit."

The fourth chapter treats of "small-pox after cow-pox," and comprises histories of four cases of what the author calls "real small-pox." In two instances the disease assumed the distinct form, in one of which it was very severe; in another instance it was confluent, and in the fourth petechial. These cases, the author properly argues, "can have no weight against the practice of vaccination, when compared with ten thousand vaccinated individuals living in the midst of a contaminated atmosphere,—with five hundred and thirty deaths amongst little more than three thousand who had neglected to be vaccinated,—and with the occasional occurrence of regular small-pox in those who formerly had the disease either naturally or by inoculation."

In the next chapter Mr. Cross gives the history "of the concomitant eruptive diseases of the epidemic small-pox." The measles "were hardly met with;" a few instances of scarlet-fever occurred during the summer; "but, upon the whole, the contagious complaints which usually occur every year amongst children, were less frequent during the epidemic."

One of the eruptive diseases which "chiefly attacked those who had neither passed through small-pox nor cow-pox," was a sort of petechial fever. It attacked but a small proportion of those who were liable to small-pox, and to them it was generally fatal. It was most common when the epidemic was at the worst. No instance of it was witnessed by the author in persons who had previously suffered small-pox or cow-pox. The victims of it were mostly children, reduced to a feeble state by scrofula or some other disease; "and, as (the author adds) several were seized with it, whilst others in the same family were suffering from small-pox, I could not help suspecting that in all it was produced by the variolous contagion.

The eruptive diseases which seemed to occur "indiscriminately in those who had passed through small-pox or cow-pox, and in those who had not had either of these disorders," were several forms of chicken-pox, which the author considers to be distinct from small-pox. His diagnostic remarks have been already considered.

The eruptive diseases which chiefly occurred in those who had passed through cow-pox, but which also, in a few instances, affected persons who had experienced small-pox, were several varieties of what has been called *modified small-pox*, which do not seem to have differed remarkably from those described by Drs. Thomson and Bent. Sixteen cases are related, the details of which must be perused, in order that any useful knowledge may be obtained from the history of them.

The second part of the work treats of the distinctive characters of the small-pox, in the first chapter; the history of varicella in the second; and then, in the next, of "modified small-pox and its classification with variola and varicella." We must refer the reader to the work itself for such of the interesting matters on these points as our limits have not permitted us to adduce. Mr. Cross presents a new classification of varioloid diseases, in which he proposes that the term *varicella* should be applied to that in which the pocks want what he considers to be the characteristics of well-defined *small-pox*. Of varicella he makes two divisions.

"1. *Varicella Cellulosa.* The fluid of the eruption contained mostly in separate cells; the pocks often depressed in the centre, but sometimes presenting only conoidal and firm vesicles placed upon a thickened basis of the cutis; incrustation taking place, without secondary fever, at various periods from the third to the seventh day; scabs mostly flat and circular, and, on falling off, leaving tubercular elevations, or convex surfaces of the cutis. It is produced by the variolous contagion, occurs sometimes after natural and inoculated small-pox, but more frequently after cow-pox. Its contagion may give rise to small-pox in those liable to that disorder, and it is capable of being inoculated, producing sometimes regular small-pox, and at others an incomplete and non-protecting disease. (*Synonyma. Stone-pock, horn-pock, modified small-pox, pemphygus variolodes solidescens, chicken-pox, varicella.*)

"2. *Varicella Bullosa.* The fluid every-where contained in vesicles composed of one cavity; the covering of the contained fluid delicate and easily broken; drying into small irregular crusts from third to fifth day, and, on falling off, leaving in every part a plain surface of the cuticle; rarely leaving pits except from scratching, when the scars are large and lighter in colour than the surrounding skin. It is doubtful if this eruption proceed from the variolous contagion; certain that it does not give rise to small-pox, and that it maintains the same character in all classes, whether occurring before or after cow-pox and small-pox; very contagious, and affecting a majority of people once during life; probably not communicable by inoculation. (*Synonyma. Crystals; water-pox; varicella; chicken-pox; pemphygus variolodes vesicularis; mild vesicular small-pox.*"

The only remarks we shall make on this division is, that we think it objectionable in the present state of our knowledge, and likely to produce confusion in historical records, if adopted; because the same generic term is applied to two forms of disease which are perhaps of different origin. The first variety, the author says, arises from the contagion of *small-pox*, whilst the second, perhaps, depends on a different specific contagious matter. If it were proved that all these forms of disease were derived from the same source, it would then be convenient that those varieties which deviate, or degenerate, from the well-marked character of the disease in its most effective form, should be designated by an epithet marking such a degeneration, as Mr. Cross here proposes.

The fourth chapter treats of the merits of small-pox inoculation, in respect to vaccination, and of the means for discouraging it. The practice of variolous inoculation, he remarks, after Sir GILBERT BLANE, tended to increase the mortality of that disease, instead of lessening it, as is shown by the following table relating to periods of fifteen years, from fifteen years previous to the use of variolous inoculation to fifteen years since vaccination has been generally established. It shows the ratio of deaths from small-pox to the whole number of deaths from all diseases in London only.

| | | | | | | |
|---|---|---|---|---|---|---|
| First period, | from | 1706 | to | 1720, | 79 | in 1000 |
| Second ditto, | — | 1745 | — | 1759, | 89 | — 1000 |
| Third ditto, | — | 1785 | — | 1798, | 94 | — 1000 |
| Fourth ditto, | — | 1805 | — | 1818, | 53 | — 1000 |

HAYGARTH, Mr. Cross shows, had observed this effect of variolous inoculation. "As far as my circle of observation extends," says Dr. H. "both in England and Wales, this improved method of communicating the distemper has manifestly appeared to be injurious to the poor, though eminently useful to the rich. It has become prejudicial to the community, though human nature never bestowed so valuable a blessing as it confers on the few intelligent individuals who adopt it. This consideration ought to have great weight; and in one of the most enlightened nations of Europe it occasioned a prohibition of inoculation in large towns, which has never been repealed." Haygarth's *Sketch of a Plan for exterminating the casual Small-Pox from Great Britain.* Introduction, page 36. (1793.)

This chapter, as well as the ensuing one, on the means of promoting vaccination, abounds too much with interesting observations and judicious suggestions to permit us to attempt to analyze them. They cannot be abridged; and probably most of our readers have already perused them.

The author has added three appendices to the work, comprising Heim's paper on the diagnosis of small-pox and varicella; the result of a correspondence with practitioners in the county of Norfolk, and adjoining parts of the county of Suffolk; and an abstract of a Report of the Vaccinations practised in France.

We have given so irregular an account of the work of Mr. Cross, and have deviated so far from the principles of analytical criticism in our review of it,—in consequence of our desire to accumulate in a small extent of space all the most essential circumstances connected with the question we have particularly examined,—that it becomes a point of duty for us to endeavour to correct the ideas our abstract is calculated to give rise to respecting it, by some general assertions. This work, then, we would have it understood, presents abundance of evidence of the same talents for accurate observation, comprehensive views, and judicious discrimination, as were displayed in the author's account of the state of medicine in Paris; and it comprises much matter that will prove both interesting and practically useful. It has the advantage, too, of being composed in a strictly methodic manner: each of the distinct views which may be taken of the subjects of it in general, is considered in a particular section, and the whole are treated of in such an order as that each series of facts serves to prepare the development and perspicuous comprehension of that which succeeds to it. We cannot say this of Dr. Thomson's work,—or, as it is, somewhat oddly, termed, his *Letter* to Sir JAMES MAC GRIGOR. The reader—unless he possesses no ordinary powers of memory and concatenation of ideas—will find it necessary to write down, under a series of heads, the chief of the propositions relating to the subject, according to which he may arrange his notes, in order that he may collect and be able to refer to the most essential facts relating to almost any one of the principal points discussed in it; they are dispersed throughout the work in so intricate a manner. There is in it, besides, a great deal of repetition. Many of the arguments are stated again and again, hardly less than twenty times, in the same general way. Dr. Abercrombie's, Dr. Alison's, and Mr. Bryce's, descriptions of chicken-pox, for example, are inserted in remote parts of the book; and, after each of them, the author goes over the same ground as that through which we had already accompanied him, producing here and there a new and important remark,—which obliges a person to peruse the whole with attention,—when he should have comprised all his principal arguments on the several distinct views of the subject in one particular series. It is this mode of writing which has extended this *letter* to above three hundred pages,—to, perhaps, at least four times the necessary space,—independant of a long Appendix containing details of cases; for the former part is constituted almost wholly of *general* observations and remarks. The consequence of this is, that but few persons have sufficient patience to read the book through, and but few of those who have read it can remember, in a connected manner, even the most important of the really interesting observations it contains.

